# Childhood emotional abuse and suicidal ideation in college students: exploring the mediating role of alexithymia and the moderating effect of physical exercise

**DOI:** 10.3389/fpsyt.2025.1660164

**Published:** 2025-11-28

**Authors:** Chang Hu, Wenying Huang, Wen Zhang

**Affiliations:** School of Physical Education, Jiangxi Normal University, Nanchang, Jiangxi, China

**Keywords:** childhood emotional abuse, alexithymia, physical exercise, college students, emotional regulation, suicidal ideation

## Abstract

**Objective:**

Childhood emotional abuse is a significant yet often overlooked predictor of suicidal ideation among young adults. This longitudinal study examined how alexithymia mediates the relationship between Childhood emotional abuse and suicidal ideation among college students, and whether physical exercise moderates both the direct and indirect paths within this association.

**Method:**

We conducted a two-wave longitudinal study (12-month interval) using convenience sampling of Chinese college students from multiple universities in Yunnan and Guizhou provinces. At T1 (April–June 2024), 1,006 valid online self-reports were collected; at T2 (April–June 2025), 926 participants were successfully matched (Mage = 20.78, SD = 2.58). Measures included childhood emotional abuse (CTQ–Emotional Abuse), alexithymia (TAS-20), suicidal ideation (PANSI), and weekly physical exercise frequency (single-item). Analyses used SPSS PROCESS (Model 4 and 15) with 5,000 bootstrap resamples, controlling for gender and birthplace.

**Results:**

The results showed that T2 alexithymia mediated the relationship between T1 childhood emotional abuse and T2 suicidal ideation. Additionally, T2 physical exercise significantly moderated the direct relationship between T1 childhood emotional abuse and T2 suicidal ideation. However, the moderating effect of physical exercise on the indirect pathway via T2 alexithymia was not significant.

**Conclusions:**

The findings highlight alexithymia as a key psychological mechanism linking emotional abuse in Childhood to suicidal ideation during emerging adulthood, while physical exercise serves as a protective factor at the direct level. Targeted interventions that enhance emotional awareness and promote regular physical activity may help mitigate suicide risk among college students with emotional abuse histories.

## 1 Introduction

Suicide has long been recognized as a critical global public health challenge, claiming nearly 800,000 lives every year worldwide (1, 2). Asian countries account for roughly two-fifths of these deaths (3, 4). It ranks as the second leading cause of mortality among people aged 15 to 29 (5). In the United States, approximately 13.9 individuals per 100,000 take their own lives annually (6), and more than one-fourth of these deaths occur among youth aged 10 to 24 (7). A similar trend has been observed in several East Asian societies. Since the early 1990s, Japan has experienced a persistent increase in suicide rates, most notably among individuals aged 15–39 (8, 9). South Korea reports more than 13,000 suicides annually, with those aged 20–39 accounting for 40% of cases (10, 11). Although China has seen a gradual decline in suicide rates overall, the incidence among younger populations remains a major concern (12, 13). Studies indicate that 15.4% of college students report suicidal ideation, and 3.5% have attempted suicide (14). According to the Three-Step Theory of suicide (15, 16), suicidal behavior typically originates from suicidal ideation, which encompasses a spectrum of self-destructive thoughts, ranging from passive wishes to die to active plans for suicide. These cognitions about death serve as a proximal indicator of suicide risk (17–19). Since suicidal thoughts often precede suicidal acts, understanding their antecedents is essential for early prevention and targeted intervention (20, 21).

The relationship between childhood emotional abuse and suicidal ideation has been well established in previous research (22, 23). However, the psychological processes that explain how early emotional abuse leads to suicidal thoughts remain insufficiently understood. Alexithymia, which reflects impairments in emotional awareness and regulation, may function as a key mediating mechanism in this association. In contrast, physical exercise, as a modifiable behavioral factor, may buffer the adverse effects of emotional abuse by fostering emotional resilience and better self-regulation. Given these considerations, the present study aims to construct a moderated mediation model to examine how childhood emotional abuse influences suicidal ideation through alexithymia and whether physical exercise moderates these relationships.

### 1.1 Suicidal ideation and childhood emotional abuse

Suicidal ideation among college students has been closely associated with adverse experiences during Childhood (24–26). Among these, childhood emotional abuse stands out as a subtle yet profoundly harmful form of trauma that has attracted increasing scholarly attention (27–29). It encompasses behaviors directed at individuals under 18 years old, including verbal degradation, emotional manipulation, or neglect of emotional needs (30). Even in the absence of physical harm, such experiences can result in enduring psychological trauma (31). Surveys indicate that approximately 38.7% of college students have experienced varying degrees of childhood emotional abuse (32, 33). Exposure to these early emotional injuries may distort self-perception and hinder the development of a coherent sense of self. Feelings of inadequacy, diminished self-worth, and emotional insecurity can amplify internal distress and hopelessness, substantially heightening vulnerability to suicidal ideation (34). From the perspective of attachment theory, the quality of early caregiver–child interactions plays a pivotal role in shaping emotional security and later psychological functioning (35). When emotional abuse occurs during sensitive developmental periods, it may severely undermine the establishment of secure attachment, which serves as a foundation for healthy emotional growth (36, 37). This erosion of attachment security often contributes to maladaptive emotional patterns, including alexithymia, characterized by difficulties in identifying, interpreting, and articulating emotions (38). Such deficits in emotional processing can have far-reaching consequences for mental wellbeing and substantially increase the risk of developing suicidal ideation (39–41).

Early research has consistently demonstrated a significant positive correlation between childhood emotional abuse and suicidal ideation among adolescents (42–45). However, most existing research has employed cross-sectional designs, lacking longitudinal evidence to capture changes and causal relationships over time. In addition, within the Chinese cultural context, emotional abuse is often misunderstood as an educational approach, with some parents even regarding verbal humiliation as a way to motivate children. This cognitive bias has led to the systematic neglect of the psychological harm caused by emotional abuse, despite its enduring negative impact on adolescents’ mental health (46).

### 1.2 Alexithymia as a mediator

How does childhood emotional abuse contribute to suicidal ideation among college students? From a psychological development perspective, childhood emotional abuse is a profound form of early trauma that can have lasting effects on psychological wellbeing (47). One critical pathway through which it influences suicidal ideation is by fostering alexithymia (48, 49). Alexithymia is understood as a relatively stable emotional-cognitive trait characterized by significant difficulties in recognizing, expressing, and communicating emotions effectively (50). Social and cultural theories emphasize that the quality of emotional interactions within the family is crucial for the development of emotional understanding (51, 52). In contrast, persistent emotional neglect or verbal hostility within the family can impede the development of a child’s emotional sensitivity and adaptive expression (53, 54). Empirical studies suggest that children subjected to emotional abuse often suppress their emotions to cope with psychological distress (55, 56). Over time, this suppression can evolve into the personality trait of alexithymia, increasing their vulnerability to suicidal ideation in adulthood (57–59).

A systematic review has identified a strong link between alexithymia and suicidal ideation (60). Hemming et al. (61) further demonstrated that individuals with limited emotional awareness and expression are more prone to suicidal impulses due to inadequate self-regulation. According to multiple code theory (62), when individuals with alexithymia encounter adverse events, they often struggle to process and express their emotions in healthy ways. Consequently, they may resort to extreme actions, such as suicide, as a means of alleviating psychological distress (63–65). Current research on the interplay between childhood emotional abuse, alexithymia, and suicidal ideation remains limited. Although Xie et al. (66) identified alexithymia as a mediator between childhood abuse and suicidal behavior among adolescents with depression, it remains unclear whether these findings apply to college student populations.

### 1.3 Physical exercise as a moderator

As the field of sports psychology continues to develop, a growing number of studies have demonstrated that physical exercise can significantly promote mental health and effectively regulate emotions (67–69). Research has shown that regular physical activity not only enhances vitality but also facilitates the release of neurotransmitters associated with emotional wellbeing, thereby reducing negative emotional experiences (70–72).

Childhood emotional abuse is strongly linked to alexithymia, a condition that impairs individuals’ ability to recognize and express emotions effectively (73). This emotional difficulty significantly increases the risk of suicidal ideation (74). However, regular physical exercise is known to enhance cognitive flexibility and strengthen emotion-regulation capacities (75, 76). For college students who have experienced emotional abuse and exhibit alexithymia, engaging in physical exercise may evoke positive emotional experiences, such as feelings of pleasure and a sense of mastery, while also promoting physical and mental relaxation. These benefits act as protective factors (77, 78) that weaken the connection between alexithymia and suicidal ideation. This perspective aligns with integrated models of athletic performance, which highlight how the cognitive, emotional, and physiological benefits of exercise create a psychological protective barrier, disrupting the adverse progression from traumatic experiences to suicidal ideation (79–81).

Within a risk–protection framework (82, 83), alexithymia serves as an important risk factor associated with childhood emotional abuse, whereas physical exercise acts as a protective factor that facilitates emotional regulation (84). College students with higher levels of exercise participation tend to manage stress more effectively, experience fewer difficulties in identifying and expressing emotions, and are less likely to translate alexithymic tendencies into suicidal ideation. In contrast, those with lower levels of exercise engagement are more prone to problematic behaviors such as depression (85), isolation (86), and restrictive eating (87), making them more vulnerable to the negative impacts of childhood emotional abuse and, consequently, at greater risk for suicidal ideation.

### 1.4 The present study

**Figure 1:**
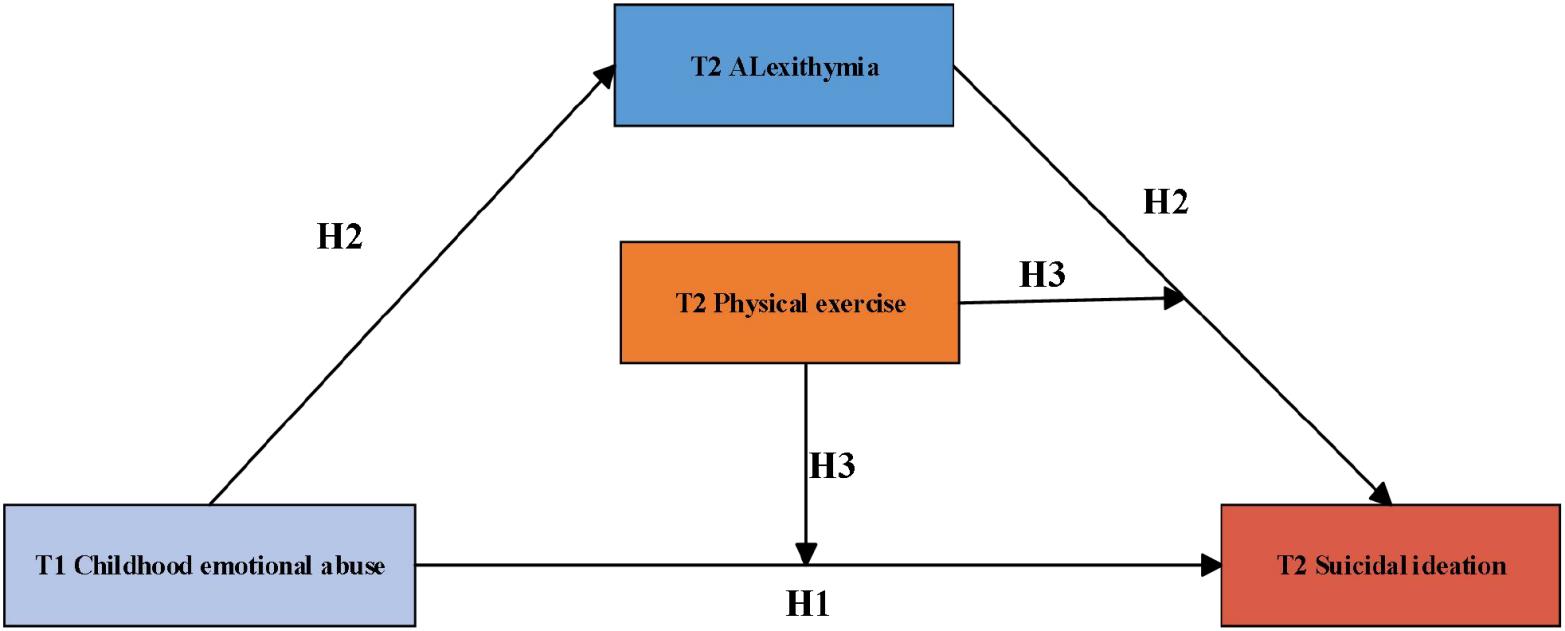
The proposed structural model.

This study aimed to develop a moderated mediation model to explore how T1 childhood emotional abuse influences T2 suicidal ideation among college students through T2 alexithymia, while examining the moderating role of T2 physical exercise in this process. A two-wave longitudinal design (T1–T2) was employed with a sample of Chinese college students, and key covariates, including gender and place of birth, were controlled for in the analyses. Grounded in attachment theory and multiple code theory, three hypotheses were proposed (1): T1 childhood emotional abuse would be positively associated with T2 suicidal ideation; (2) T2 alexithymia would mediate the association between T1 childhood emotional abuse and T2 suicidal ideation; (3) T2 physical exercise would moderate both the direct association between T1 childhood emotional abuse and T2 suicidal ideation, and the indirect association via T2 alexithymia. The hypothesized model is presented in **Figure 1**. Moreover, given evidence that gender and place of birth may influence the focal variables, these factors were included as covariates in all analyses.

## 2 Methods

### 2.1 Participants and procedures

This study utilized a two-wave longitudinal design and recruited currently enrolled college students from four universities in Southwest China (Guizhou and Yunnan provinces) using convenience sampling. Questionnaires were distributed and collected by faculty teams in physical education departments at partner universities through WeChat/QQ, the most widely used social platforms in China. Data were collected at two time points, 12 months apart.

At T1 (April–June 2024), a total of 1,118 questionnaires were received, of which 1,006 were valid (validity rate = 89.98%). At T2 (April–June 2025), 982 participants completed the follow-up, and after quality control, 56 invalid questionnaires were excluded. The final longitudinal sample consisted of 926 participants who completed both waves, corresponding to an attrition rate of 7.95% from T1 to T2. To enable tracking and matching, the last four digits of mobile phone numbers and the first four digits of student IDs were collected as identifiers with the participants’ consent and stored separately from survey responses. During the second wave, three rounds of reminders were sent to minimize participant attrition. All data collection and processing adhered to research ethics and data protection protocols and were used exclusively for academic purposes.

Inclusion criteria were: full-time enrolled students; no systematic psychotherapy or psychiatric medication use in the past six months (self-reported); provision of electronic informed consent; and completion of the online questionnaires at both T1 and T2. Exclusion criteria included: completion time below the threshold (<180 seconds); mismatches in the last four digits of the mobile phone number or the first four digits of the student ID used for matching between T1 and T2; patterned responding with identical options across all items within any core scale; and more than 20% missing data on key variables. The primary analyses used complete cases (n = 926 with valid data at both T1 and T2). To reduce measurement bias and invalid responses, we employed standardized instructions and unified administration procedures, embedded attention-check items (e.g., “Please select ‘Strongly disagree’”), randomized item order, and enabled system-level settings that limit submissions to 1 per IP address and disallow blank submissions.

At T1, the final sample comprised 926 participants: 515 men (55.6%) and 411 women (44.4%). By birthplace, 461 participants (49.8%) were from rural areas, and 465 (50.2%) were from urban areas. Regarding educational levels, the sample comprised 803 undergraduates (86.7%), 101 master’s students (10.9%), and 22 doctoral students (2.4%). The mean age was 20.775 years (SD = 2.576).

Participants received an electronic incentive of 2 RMB (approximately $0.28) upon completing the T1 survey and an additional 5 RMB (approximately $0.69) for completing T2. All participants were informed that the study was conducted under strict confidentiality, that their personal information would be protected, and that they could withdraw from participation at any time. The study complied with the ethical principles outlined in the Declaration of Helsinki and was approved by the institutional ethics committee.

### 2.2 Measures

#### 2.2.1 Childhood emotional abuse (T1)

Childhood emotional abuse was evaluated using the 5-item Emotional Abuse subscale of the Childhood Trauma Questionnaire-Short Form (88). Participants were asked to report their experiences during Childhood (e.g., being called names such as “stupid,” “lazy,” or “ugly”) on a 5-point Likert scale, ranging from 1 (Never) to 5 (Almost Always). This instrument has been widely applied in studies involving Chinese college students (89, 90) and demonstrated adequate reliability in the current sample (Cronbach’s α = 0.809).

#### 2.2.2 Alexithymia (T2)

The 20-item Toronto Alexithymia Scale (TAS-20) was used to measure alexithymia (91). This scale includes three subscales, with items such as “I often cannot identify what emotion I am feeling right now.” Responses were collected on a 5-point Likert scale (1 = Strongly Disagree to 5 = Strongly Agree), with higher total scores reflecting more severe alexithymia. The TAS-20 has been validated in Chinese college student populations (92, 93) and showed strong internal consistency in this study (Cronbach’s α = 0.835).

#### 2.2.3 Suicidal ideation (T2)

Suicidal ideation was assessed using the 14-item Positive and Negative Suicidal Ideation Scale (PANSI) (94). This measure consists of two subscales, with items such as “I have thoughts about ending my life.” Participants responded on a 5-point Likert scale, ranging from 1 (Never) to 5 (Always), where higher scores indicated greater severity of suicidal ideation. The Positive Suicidal Ideation subscale was reverse-scored. This scale has been frequently utilized with Chinese college students (95, 96) and demonstrated excellent internal consistency in this sample (Cronbach’s α = 0.910).

#### 2.2.4 Physical exercise (T2)

Physical exercise was measured using a single-item question: “In the past seven days, on how many days did you engage in at least 20 minutes of exercise or activity that made you sweat or breathe hard?” Responses ranged from 0 to 7 days and were treated as a continuous variable in the primary analyses. We chose this brief question format to reduce participant burden, minimize attrition and measurement fatigue, and thereby ensure the feasibility of the longitudinal study. This single-item screening approach is widely used in health behavior research to roughly differentiate higher versus lower levels of moderate-to-vigorous activity (97, 98), and similar questions have been applied in studies of Chinese university students (99–101). However, this measure does not capture exercise duration beyond 20 minutes, detailed intensity spectrum, within-day frequency, or activity type. To evaluate the robustness of the results, we dichotomized participants at the median of 3 activity days per week (≤3 versus >3) and reran the moderation analyses.

#### 2.2.5 Covariates

To ensure the robustness of our findings, we controlled for two key demographic variables that may influence the primary constructs under investigation (102, 103). Gender was dummy-coded (0 = female, 1 = male) to account for potential sex-based differences in psychological outcomes. Birthplace was similarly dummy-coded (0 = rural, 1 = urban).

### 2.3 Data analysis

All statistical analyses were conducted using SPSS 26.0. Continuous variables were characterized by their means and standard deviations, while frequencies and percentages summarized categorical variables. To begin, Pearson correlation coefficients were calculated among the key variables, including T1 childhood emotional abuse, T2 alexithymia, T2 suicidal ideation, and T2 physical exercise. Prior to the analyses, all variables were standardized to ensure comparability and consistency across measures. For the mediation analysis, we employed the PROCESS macro (Model 4), with T1 childhood emotional abuse as the independent variable, T2 alexithymia as the mediator, and T2 suicidal ideation as the dependent variable. To examine the moderated mediation effects, we utilized the PROCESS macro (Model 15), which tested whether T2 physical exercise moderated the relationship between T2 alexithymia and T2 suicidal ideation, as well as the direct relationship between T1 childhood emotional abuse and T2 suicidal ideation. Gender and birthplace were included as control variables in all analyses. All PROCESS models were based on 5,000 bootstrap resamples to generate bias-corrected 95% confidence intervals (CIs). Effects were considered statistically significant if the CIs did not include zero. For further interpretation, simple slopes analyses were conducted and visualized at ±1 SD of physical exercise, illustrating the moderating effect.

## 3 Results

### 3.1 Preliminary analysis

Preliminary analyses revealed significant positive correlations among childhood emotional abuse, alexithymia, and suicidal ideation (*ps* < 0.01), whereas physical exercise was negatively associated with all three variables (see **Table 1**). Furthermore, variance inflation factor values for all predictors (1.12–1.28) were well below the conventional cutoff of 10 (104), suggesting no multicollinearity concerns.

**Table 1:** Descriptive statistics and pearson correlations among key study variables.

Variables	*M±SD*	Skewness	Kurtosis	1	2	3	4
1. T1 Childhood Emotional Abuse	2.169 ± 0.749	1.605	2.781	1			
2. T2 Alexithymia	2.685 ± 0.664	0.234	-0.424	0.277**	1		
3. T2 Suicidal Ideation	2.105 ± 0.697	1.944	3.389	0.471**	0.462**	1	
4. T2 Physical exercise	3.264 ± 1.974	0.129	-0.992	-0.282**	-0.433**	-0.511**	1

**p <0.01 (two-tailed). T1 = Time 1; T2 = Time 2.

### 3.2 Testing for the mediation effect

The mediation model was tested using the PROCESS macro (Model 4) with bias-corrected percentile bootstrap resampling. Controlling for gender and birthplace, the results indicated that T1 childhood emotional abuse was positively associated with T2 alexithymia (*β* = 0.259, *t* = 8.292, *p* < 0.001) and with T2 suicidal ideation (*β* = 0.373, *t* = 13.409, *p* < 0.001). Additionally, T2 alexithymia was positively associated with T2 suicidal ideation (*β* = 0.370, *t* = 13.047, *p* < 0.001).

Based on 5,000 bootstrap resamples, the indirect effect of T1 childhood emotional abuse on T2 suicidal ideation via T2 alexithymia was significant (indirect effect = 0.096, 95% CI [0.066, 0.126]), accounting for 20.47% of the total effect. The model explained 11.40% of the variance in T2 alexithymia and 34.40% of the variance in T2 suicidal ideation.

### 3.3 Testing for the moderated mediation effect

The moderated mediation model was tested using the PROCESS macro (Model 15), controlling for gender and birthplace (see **Table 2**). The results showed that T2 physical exercise significantly moderated the association between T1 childhood emotional abuse and T2 suicidal ideation (*β* = -0.224, *t* = -9.960, *p* < 0.001). However, the moderating effect of T2 physical exercise on the relationship between T2 alexithymia and T2 suicidal ideation was not significant (*β* = 0.001, *t* = 0.051, *p* > 0.05).

**Table 2:** Moderation effect regression results.

Variables	T2 Alexithymia		T2 Suicidal ideation	
	*β*	*t*	*β*	*t*
Gender	-0.386	-6.174^***^	0.064	1.305
Birthplace	0.071	1.139	-0.026	-0.550
T1 Childhood emotional abuse	0.259	8.292^***^	0.269	10.414**
T2 Alexithymia			0.210	7.667***
T1 Childhood emotional abuse× T2 Physical exercise			-0.224	-9.960 ***
T2 Alexithymia × T2 Physical exercise			0.001	0.051
*R^2^*	0.114		0.484	
*F*	39.486***		122.877***	

*p<0.05, **p <0.01, ***p <0.001.

**Figure 2:**
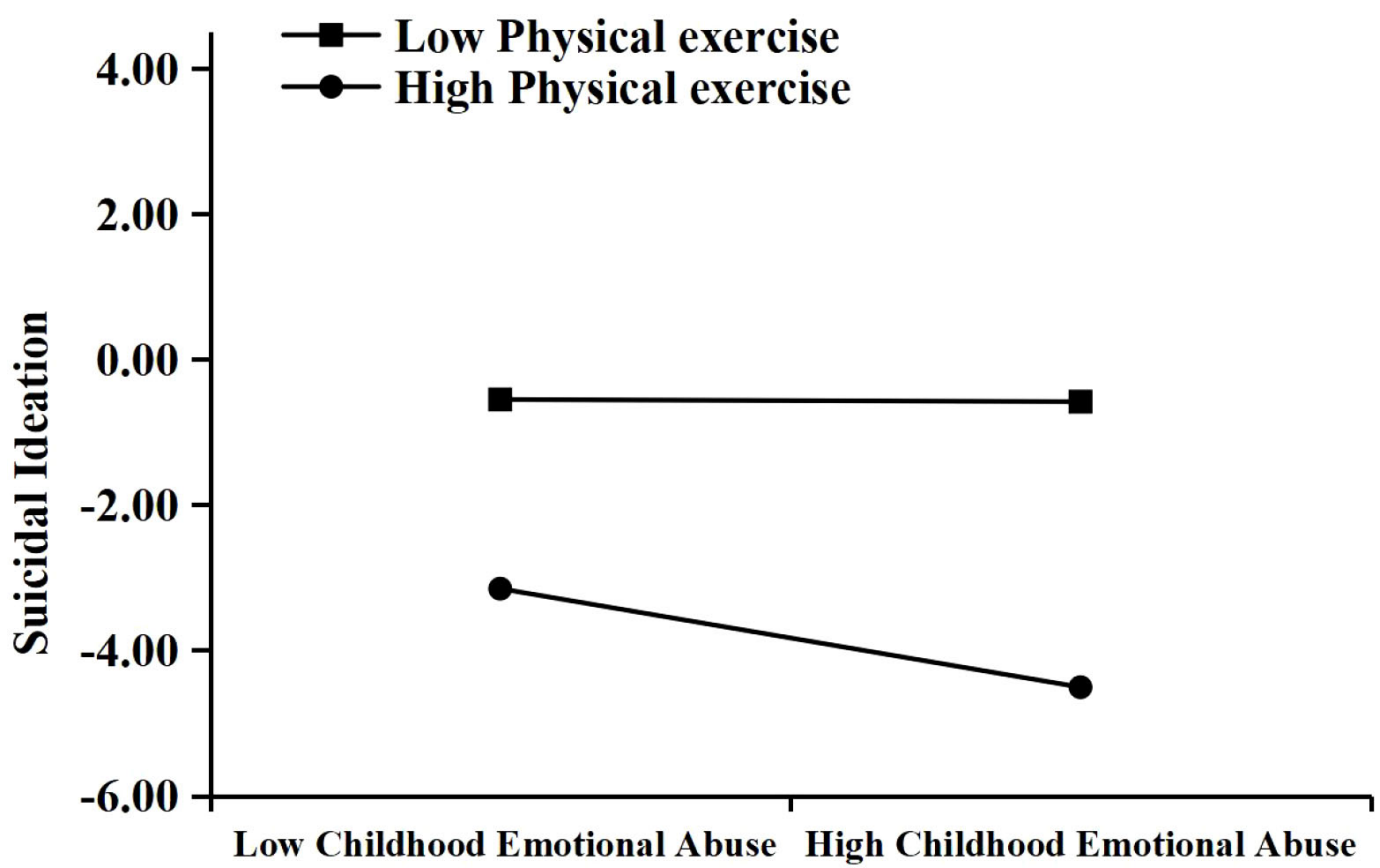
Simple slopes illustrating the moderating effect of T2 physical exercise on the association between T1 childhood emotional abuse and T2 suicidal ideation.

To further clarify the moderated mediation model, participants were categorized into high and low physical exercise groups based on ±1 SD around the T2 physical exercise mean, and simple slope analyses were performed (see **Figure 2**). The results indicated that in the low physical exercise group, the association between T1 childhood emotional abuse and T2 suicidal ideation was stronger and significant (*β* = 0.493, *t* = 15.540, *p* < 0.001). In contrast, in the high physical exercise group, this association was not significant (*β* = 0.045, *t* = 1.234, *p* > 0.05).

To address potential limitations of the single-item measure of physical exercise, we conducted a robustness check following Hu et al. (99). Participants were divided into two groups based on the sample median of physical activity (median = 3): those with scores ≤ 3 were categorized as the low physical exercise group, and those with scores ≥ 4 as the high physical exercise group. The moderation analysis under this grouping yielded results consistent with the primary findings: the interaction between T2 physical exercise and T1 childhood emotional abuse remained significant (*β* = -0.484, *t* = -9.070, *p* < 0.001), further supporting the buffering role of physical exercise.

## 4 Discussion

### 4.1 Suicidal ideation and childhood emotional abuse

The findings support Hypothesis 1, revealing a significant positive correlation between T1 childhood emotional abuse and T2 suicidal ideation. This suggests that childhood emotional abuse has a direct and profound negative impact on the mental health of college students, significantly increasing the risk of suicidal ideation. Manifested as verbal degradation, emotional neglect, or manipulation, emotional abuse undermines personal factors such as self-esteem and emotional regulation. It also weakens an individual’s sense of security and trust in others, and fosters feelings of isolation and helplessness. These effects, in turn, reduce the individual’s ability to cope with stress during the college years. This aligns with the mechanism of interaction among behavior, personal factors, and the environment proposed by Bandura’s Triadic Reciprocal Causation Theory (105).

Moreover, according to Bucci’s multiple code theory (62), prolonged emotional abuse disrupts emotional representation and processing systems. This makes it more difficult for individuals to manage psychological pain healthily when faced with academic or interpersonal stress. In such situations, suicidal ideation may be subjectively perceived as a “means of relief” from emotional suffering. In the Chinese cultural context, emotional abuse is often misinterpreted as “strict discipline.” This can involve humiliating or harsh verbal criticism intended to motivate children to excel (46). However, this approach neglects children’s emotional needs and can result in cumulative psychological trauma. This trauma becomes particularly pronounced during college, a critical stage of psychological development. These findings are consistent with previous research, which has shown that childhood emotional abuse depletes psychological resources, thereby increasing the risk of suicidal ideation in adulthood (53, 66, 106).

### 4.2 The mediating role of alexithymia

The findings support Hypothesis 2, indicating that T2 alexithymia mediated the association between T1 childhood emotional abuse and T2 suicidal ideation, such that childhood emotional abuse exerted an indirect effect on suicidal ideation via alexithymia. Emotional abuse impairs the development of emotional cognition and expression, fostering emotional shutdown and deficits in identifying, understanding, and expressing emotions; these deficits are manifested as alexithymia, marked by unclear self-perceptions, interpersonal difficulties, and poor emotion regulation (28, 107–109). Such disruptions undermine healthy emotional processing and heighten vulnerability to psychological problems and suicidal ideation (110, 111). Consistent with trauma-stress theory, emotion regulation deficits are a key driver of suicidal ideation (112); individuals with alexithymia struggle to process and release distress adaptively, so under academic pressure or interpersonal conflict, they may perceive suicide as a way to escape suffering (48, 61). In Chinese family contexts, strong emphasis on academic achievement and family reputation can translate into excessive control, criticism, or neglect of emotional needs, unintentionally reinforcing emotional suppression and alexithymia, which over time elevates college students’ risk of suicidal ideation.

### 4.3 The moderating role of physical exercise

The findings partially supported Hypothesis 3. T2 physical exercise significantly moderated the direct association between T1 childhood emotional abuse and T2 suicidal ideation, but did not moderate the indirect association via T2 alexithymia. In other words, exercise serves as a protective factor along the direct path from childhood emotional abuse to suicidal ideation, whereas it does not alter the mediating effect of alexithymia along the indirect path from childhood emotional abuse to suicidal ideation.

Along the direct path, higher levels of physical exercise attenuated the link between childhood emotional abuse and suicidal ideation. Exercise provides positive emotional experiences, improves physiological functioning, and strengthens psychological resilience. Through neurotransmitters such as endorphins, it improves mood, and, with sustained engagement, it enhances self-efficacy and coping skills, thereby reducing the risk of suicidal ideation (67–72). Along the indirect path, regardless of exercise participation, alexithymia continued to significantly mediate the association between childhood emotional abuse and suicidal ideation, suggesting that alexithymia plays a relatively stable, trait-like role that is not easily modified by exercise in the short term. By contrast, the emotional distress directly linked to childhood emotional abuse appears more state-like and more responsive to exercise-induced affect regulation (113, 114).

### 4.4 Strengths and limitations

This study has two main strengths. First, the two-wave longitudinal design reduces retrospective memory bias and avoids the interpretive limitations of cross-sectional research, thereby enhancing internal validity. Second, within the frameworks of attachment theory and multiple code theory, we constructed a moderated mediation model that elucidates how childhood emotional abuse influences suicidal ideation via alexithymia and clarifies the moderating role of physical exercise. Building on this, we advance existing theoretical models in four ways (1). Strengthening causal direction with temporal evidence: compared with prior cross-sectional studies (66, 74), our findings show that T1 emotional abuse measured predicts T2 suicidal ideation both directly and indirectly via T2 alexithymia, providing stronger support for the sequence from emotional abuse to alexithymia to suicidal ideation. (2) Differentiating trait versus state mechanisms: physical exercise buffered only the direct path from emotional abuse to suicidal ideation but not the indirect path via alexithymia, suggesting that exercise primarily targets state-like distress and stress reactivity, whereas its impact on the more stable representational and processing deficit of alexithymia is limited. This selective buffering refines the risk–protection framework and aligns with multiple code theory regarding the relative stability of translation deficits from somatic and image systems to verbal and symbolic systems. (3) Extending contextual and sample boundaries: we replicate and extend the mediating role of alexithymia in a non-clinical university sample, supporting its cross-sample robustness, and we show that, in the Chinese cultural context, normalized strict or shaming discipline may constrain the socialization of emotional representation and expression, reinforcing the development and maintenance of alexithymia. This provides culturally attuned evidence for attachment and emotion socialization theories. (4) Layering intervention targets: by juxtaposing a malleable behavioral protective factor (exercise) with a relatively stable emotional–cognitive trait (alexithymia) in one model, we propose a tiered intervention pathway. In the short term, exercise and emotion regulation training can buffer direct risk; in the longer term, psychoeducation and expressive interventions targeting alexithymia can remediate representational and symbolization deficits. This tighter linkage between risk–protection and emotion-processing theories and actionable strategies enhances theoretical utility.

Despite these strengths, several limitations should be noted and addressed in future research. First, the measurement of physical exercise relied on a single-item indicator, which may not fully capture the multidimensional nature of exercise behavior, including frequency, duration, intensity, and exercise type. Such a simplified approach might lead to biased interpretations of the psychological mechanisms underlying physical exercise. Future studies should employ multidimensional and objective assessments such as validated physical activity questionnaires, accelerometers, or wearable devices to obtain more accurate and dynamic behavioral data and examine how different dimensions of physical exercise contribute to psychological regulation.

Second, although the two-wave longitudinal design helped reduce recall bias and allowed a preliminary analysis of temporal relationships, it remains insufficient for revealing long-term dynamic interactions among the variables. Future research may consider employing multi-wave longitudinal designs, cross-lagged panel models, or latent growth models to depict the developmental trajectories and reciprocal influences of childhood emotional abuse, alexithymia, and suicidal ideation more precisely.

Third, the sample consisted solely of college students from Southwest China, which limits the generalizability of the findings. Regional and cultural differences in parenting practices, social support structures, and norms surrounding emotional expression may shape both the manifestation and consequences of childhood emotional abuse. Future studies should expand sample diversity by including participants from various regions, age groups, and clinical populations, as well as conducting cross-cultural research to explore both the universality and the cultural specificity of emotional abuse and emotion regulation mechanisms.

Fourth, only gender and birthplace were used as control variables, leaving out other potential influences. Future studies should incorporate additional demographic, familial, and psychological factors such as socioeconomic status, parental education, family functioning, and perceived social support to better account for potential confounders. Psychological variables such as depression, anxiety, self-esteem, and coping strategies may also operate as mediators or moderators between childhood emotional abuse and suicidal ideation and thus merit further investigation.

Fifth, all study variables were assessed using self-report questionnaires, which may have introduced biases such as social desirability, recall distortion, and common method variance. Considering the sensitivity of constructs such as childhood emotional abuse and suicidal ideation, future research could adopt multi-method data collection approaches to enhance reliability and validity. Integrating self-reports with teacher or peer evaluations, semi-structured clinical interviews, behavioral observations, or physiological indicators (e.g., cortisol levels, heart rate variability) would help increase objectivity and ecological validity.

Lastly, this study did not examine potential biological mechanisms, particularly epigenetic processes, that may link early adversity to suicide risk. Recent evidence suggests that childhood emotional abuse may contribute to stress-related biological aging by altering biomarkers such as telomere length or mitochondrial DNA copy number (115, 116). Future research could integrate psychological and biological data to explore biopsychosocial pathways, thereby offering a more comprehensive understanding of how emotional abuse exerts long-term effects and providing new perspectives on the cross-level mechanisms underlying post-traumatic psychological vulnerability (117).

### 4.5 Implications

The present findings extend the understanding of how early adverse emotional experiences shape suicidal ideation in young adulthood and further highlight potential avenues for prevention and intervention within university settings. Building on these results, several practical implications can be proposed to reduce suicide risk among college students.

To begin with, it is essential to prioritize the identification of college students who have experienced childhood emotional abuse. University mental health service systems should establish targeted screening and intervention mechanisms that incorporate a history of childhood emotional abuse into routine psychological assessments. Specifically, potential high-risk individuals can be identified during orientation programs for new students or through annual mental health surveys, using a combination of standardized questionnaires and individual interviews. Based on the screening outcomes, a tiered and differentiated psychological support system should be implemented. For students at higher risk, counseling services grounded in trauma-informed care can be provided, focusing on rebuilding a sense of safety, strengthening emotional recognition skills, and enhancing self-worth to help repair the emotional regulation and trust mechanisms damaged by early adverse experiences. For students at relatively lower risk but who exhibit emotional suppression or difficulty in emotional expression, group-based developmental programs such as Emotional Awareness and Expression Workshops or Self-Compassion Training can be organized to promote emotional awareness and regulation. In addition, universities should actively collaborate with external professional institutions or research teams to jointly develop specialized intervention programs targeting trauma recovery and emotional functioning restoration. By establishing an integrated mental health service network that encompasses screening, intervention, follow-up, and evaluation, universities can more effectively mitigate the long-term psychological impact of childhood emotional abuse and better support students’ mental wellbeing.

Moreover, addressing difficulties in emotional regulation is equally critical, given that alexithymia serves as a key psychological pathway linking childhood emotional abuse to suicidal ideation. Campus-based mental health professionals should develop and implement psychoeducational programs and skill-training interventions aimed at enhancing students’ emotional awareness, expression, and regulation. Workshops or courses focusing on emotion identification, cognitive-emotional adjustment, and mindfulness-based practices can help students recognize, label, and communicate their feelings more effectively. Empirical evidence has demonstrated that structured emotional regulation training can significantly reduce alexithymia and psychological distress among young adults, thereby lowering suicide risk (118). In addition, strengthening peer-support networks and improving access to on-campus psychological resources are essential for creating an emotionally supportive environment. Student organizations, educators, and counseling centers can collaborate to organize peer mentoring programs, social and community-building activities, and mental health campaigns. These initiatives help foster interpersonal connectedness and reduce students’ sense of isolation. Cultivating a campus culture characterized by empathy, openness, and inclusion can further buffer the adverse psychological effects of early emotional trauma and improve overall emotional wellbeing among college students.

Lastly, promoting regular physical exercise among college students is a crucial strategy for enhancing psychological resilience and reducing the risk of suicidal ideation. Regular physical activity not only improves physical health but also provides significant psychological benefits by promoting the release of dopamine and endorphins, enhancing sleep quality, and stabilizing mood. These benefits contribute to better emotion regulation, stress relief, and overall psychological wellbeing. For college students with a history of childhood emotional abuse, engaging in physical exercise can effectively reduce symptoms of depression and anxiety and lower the risk of suicidal ideation. Given these benefits, universities should systematically integrate physical activity into their mental health promotion programs, creating a comprehensive intervention model that combines physical and mental health. Mental health service centers can collaborate with physical education departments to offer mindfulness-based exercise programs such as Emotion Regulation Yoga, Mindful Running Groups, or Mind-Body Resilience Camps. These programs can help students enhance their body awareness and emotion regulation skills. To increase student participation and adherence, universities can implement exercise tracking platforms and incentive mechanisms that reward regular physical activity. Encouraging students to form sports clubs or outdoor activity groups can also foster positive social connections and reduce feelings of loneliness. By developing a campus culture that values physical activity, universities can enhance students’ self-efficacy and psychological resilience, providing a practical and scalable approach to mitigating the long-term psychological risks associated with childhood emotional abuse and promoting overall mental health.

## 5 Conclusions

This two-wave longitudinal study found that childhood emotional abuse significantly predicted suicidal ideation among college students, with alexithymia acting as a partial mediator. Physical exercise significantly moderated the direct relationship between childhood emotional abuse and suicidal ideation but not the indirect pathway through alexithymia.

## Data Availability

The original contributions presented in the study are included in the article/Supplementary Material. Further inquiries can be directed to the corresponding author.
